# Deltopectoral flap revisited for reconstruction surgery in patients with advanced thyroid cancer: a case report

**DOI:** 10.1186/s12893-017-0297-8

**Published:** 2017-09-15

**Authors:** Taro Mikami, Shintaro Kagimoto, Yuichiro Yabuki, Kazunori Yasumura, Toshinori Iwai, Jiro Maegawa, Nobuyasu Suganuma, Shohei Hirakawa, Katsuhiko Masudo

**Affiliations:** 1 Department of Plastic and Reconstructive Surgery, Yokohama City University Hospital, 236-0004 Kanazawa-ku, Yokohama city, Kanagawa prefecture Japan; 2 Department of Oral and Maxillofacial Surgery, Yokohama City University Graduate School of Medicine, Yokohama, Japan; 3 Department of General Surgery, Yokohama City University Hospital, Yokohama, Japan

**Keywords:** Thyroid cancer, Deltopectoral flap, Reconstruction, Neck dissection, Advanced stage

## Abstract

**Background:**

We present the cases of 2 patients with invasive thyroid cancer, who underwent reconstructive surgery using a deltopectoral flap. Although the overall rate of extrathyroidal extension in patients with thyroid cancer is quite low, skin invasion is the most common pattern observed. Reconstructive surgery, involving local skin flaps, is required in these patients. The deltopectoral flap relies on the blood supply from intercostal perforators of the internal thoracic artery and usually requires skin grafting to the donor site. The internal thoracic artery is rarely sacrificed in these cases, even in an advanced surgery such as in patients with invasive thyroid cancer.

**Case presentation:**

A 55-year-old man with a distended thyroid gland presented to our hospital. He underwent advanced surgery, including skin excision, because we suspected that his tumor was thyroid cancer. The defect was covered with an ipsilateral deltopectoral flap via transposition of the flap, without skin grafting. In the second case, a 67-year-old woman with thyroid cancer that metastasized to her neck lymph nodes presented to our institution. Although the ipsilateral internal thoracic artery was sacrificed near its origin during tumor resection, the deltopectoral flap was raised in the usual manner without any complications. The skin defect caused by the tumor resection was covered with the flap. The patient had an uneventful clinical course for more than 2 years of follow-up.

These 2 cases show the effectiveness of using the deltopectoral flap as a reconstructive option for patients with thyroid cancer who underwent radical surgery, resulting in a skin defect. The first case shows that this flap does not always require skin grafting to the donor site. To our knowledge, the second case may be the first report of a deltopectoral flap that was safely raised and applied with resection of the bifurcation of the ipsilateral internal thoracic artery.

**Conclusions:**

Although thyroid cancer surgery with surrounding skin excision is a rare procedure, we found that the deltopectoral flap was useful and should be the first choice for patients undergoing reconstructive surgery, whether the bifurcation of the ipsilateral internal thoracic artery is sacrificed.

**Electronic supplementary material:**

The online version of this article (10.1186/s12893-017-0297-8) contains supplementary material, which is available to authorized users.

## Background

The incidence of thyroid cancer is relatively low in Japan—13 per 10 million individuals—however, it is of particular interest now because of the recent accidents involving nuclear power plants, including those in Fukushima, not only in this country, but also in other countries [1–3].

Although skin invasion is the most common pattern of extrathyroidal extension in patients with thyroid cancer, the overall rate of invasion is only 4%, even in well-differentiated adenocarcinoma [4]. Therefore, there are only a few reports of reconstruction in advanced thyroid cancer cases.

In the past 10 years, only 2 of approximately 600 cases of thyroid cancer surgery performed at our institution included reconstructive surgery; both cases involved invasion of the skin from the neck. A deltopectoral (DP) flap, a well-known flap used in reconstructive surgery, was applied in both cases using the standard technique. One case provided new information concerning blood flow of the DP flap, and the second one, an example of irregular treatment of the donor site.

## Case presentation

### Patient 1

**Fig. 1:**
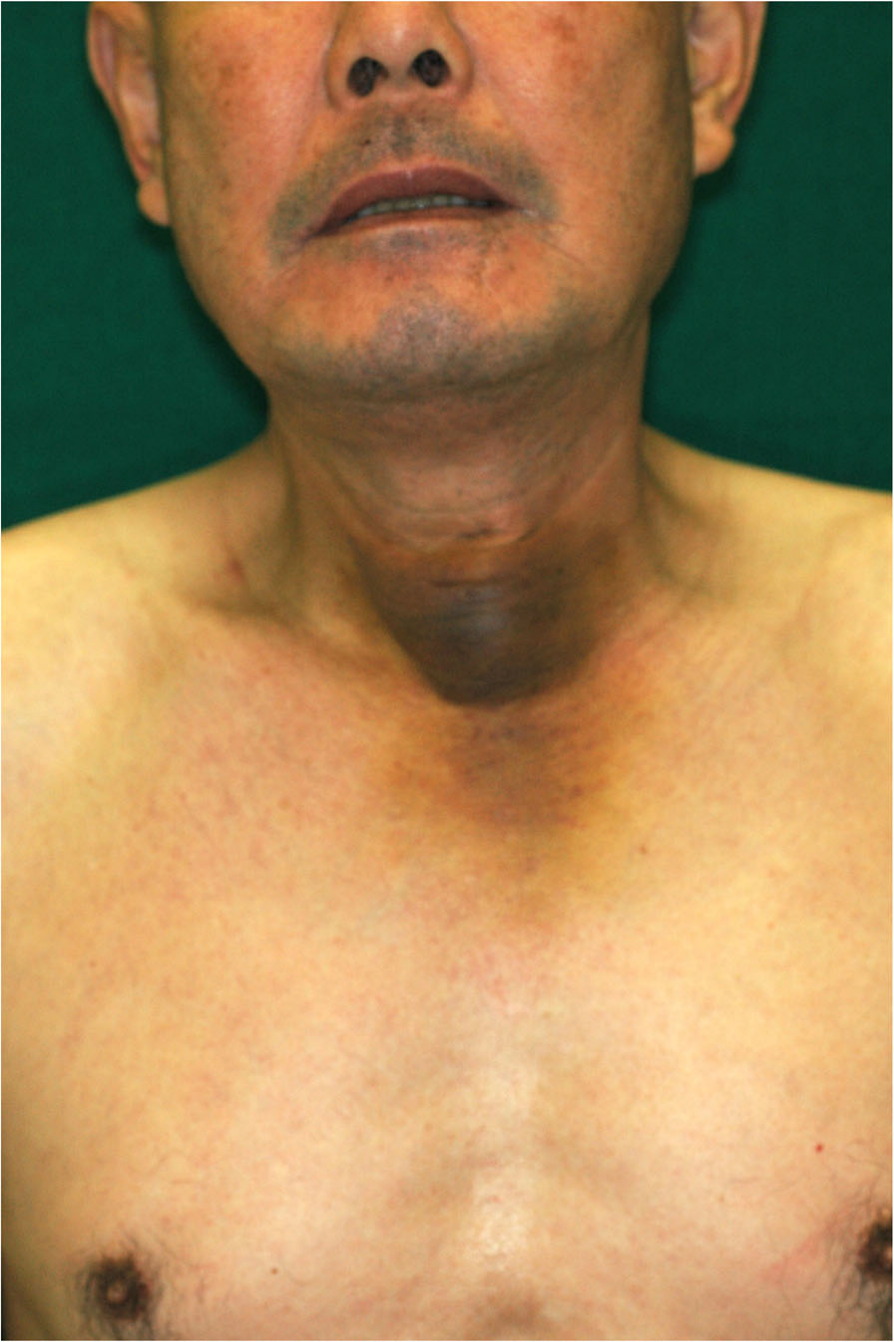
Preoperative findings of case 1. The lower neck shows obvious swelling. The skin is pigmented, presumably due to inflammation caused by thyroid cancer

**Fig. 2:**
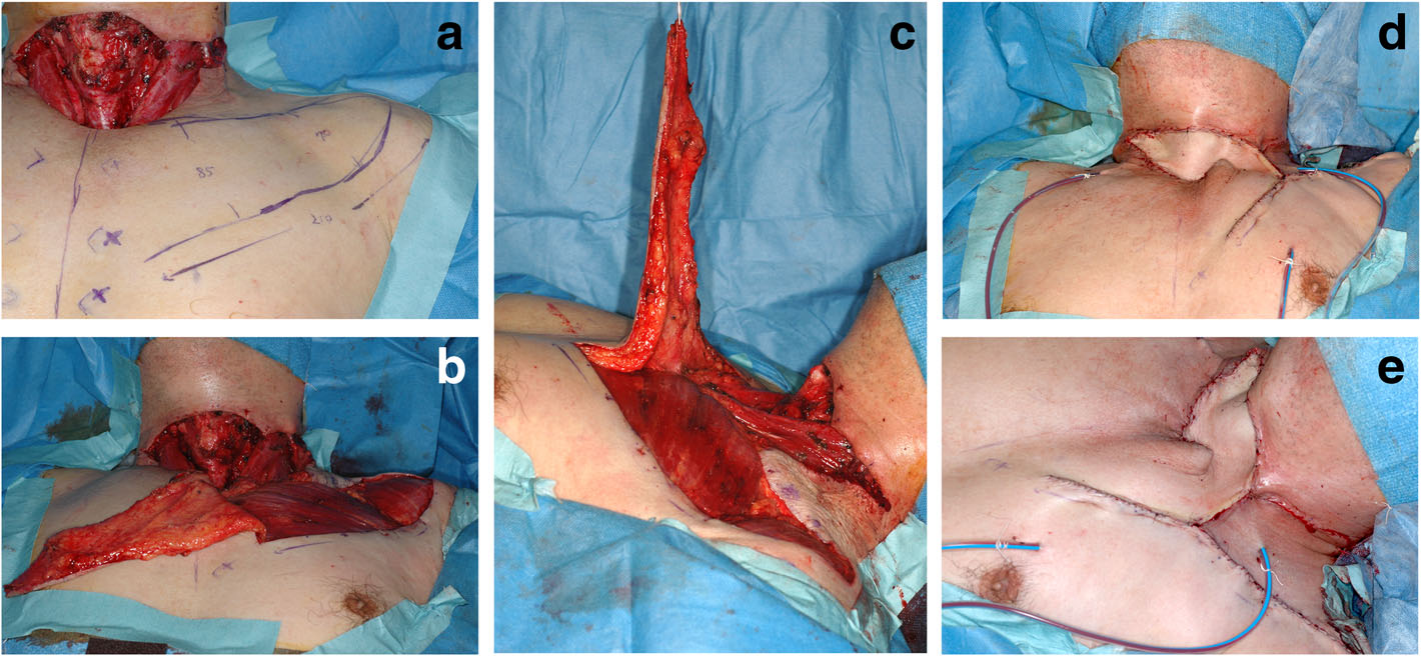
The DP flap design and dimensions of case 1. **a** The design and landmarks were drawn on the patient’s skin, using gentian violet. Previously, the thyroid gland was entirely removed, followed by bilateral neck dissection. The DP flap included the 2nd and 3rd intercostal perforator vessels, as per the usual technique, which was thought to provide adequate blood supply for the flap in this case. **b**, **c** Dimensions of the flap. As the surface of the pectoralis major and the deltoid muscle can be seen, the DP flap is confirmed as a fascio-cutaneous flap, as is usually found. **d**, **e** Final patient outcome. The donor site of the flap was closed directly by undermining the wound edges of the incision for left neck dissection. Suction drains were fixed without air leak

**Fig. 3:**
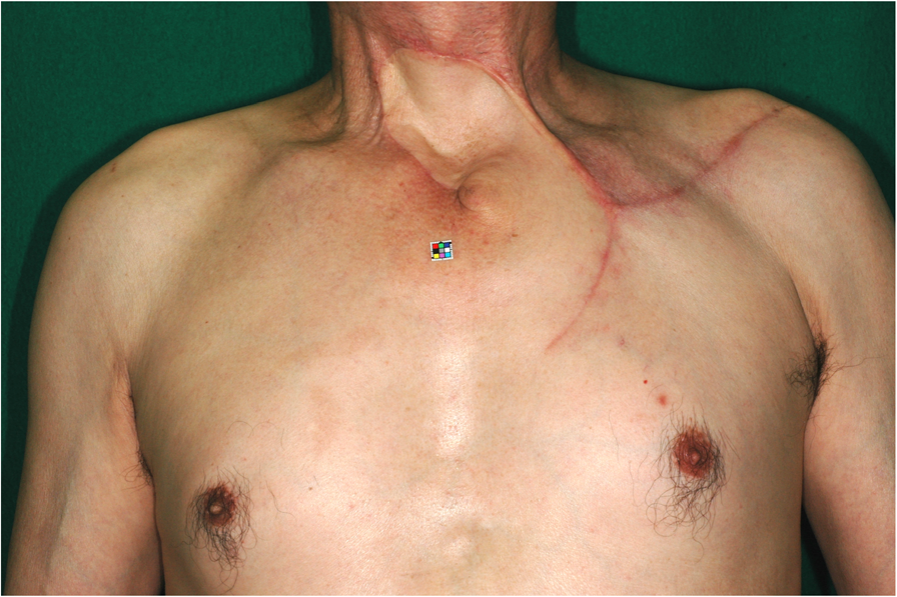
Local findings of case 1 at postoperative 6 months. There is no obvious deformity, except for upper deviation of the left nipple. A mild scar contracture is observed around the left clavicle, which was revised 15 months after primary surgery by performing multiple z-plasty

A 55-year-old man presented to our hospital with suspected carcinoma of the thyroid gland. The tumor in the center of the neck was large enough for us to suspect that the skin invasion had been present for many years, although the patient had no remarkable symptoms. Based on needle biopsy findings, the tumor was diagnosed as papillary carcinoma. In addition to total thyroidectomy, we planned for an optional skin excision with bilateral neck dissection based on the CT findings (Fig. 1). We originally planned to use a local flap of the neck to cover the skin defect, but the size of the defect was too large and we had to make an extended skin incision (Fig. 2a). A DP flap was designed with substantial undermining to the pectoral region, followed by direct closure of the surgical wound and donor site, using suction drains and without a skin graft (Fig. 2b-d). No adverse events occurred during hospitalization. The patient initially declined multiple z-plasty 6 months after the initial surgery to treat the scar contracture around his neck, but later accepted our recommendation (Fig. 3 and Additional file 1: Figure S1).

### Patient 2

**Fig. 4:**
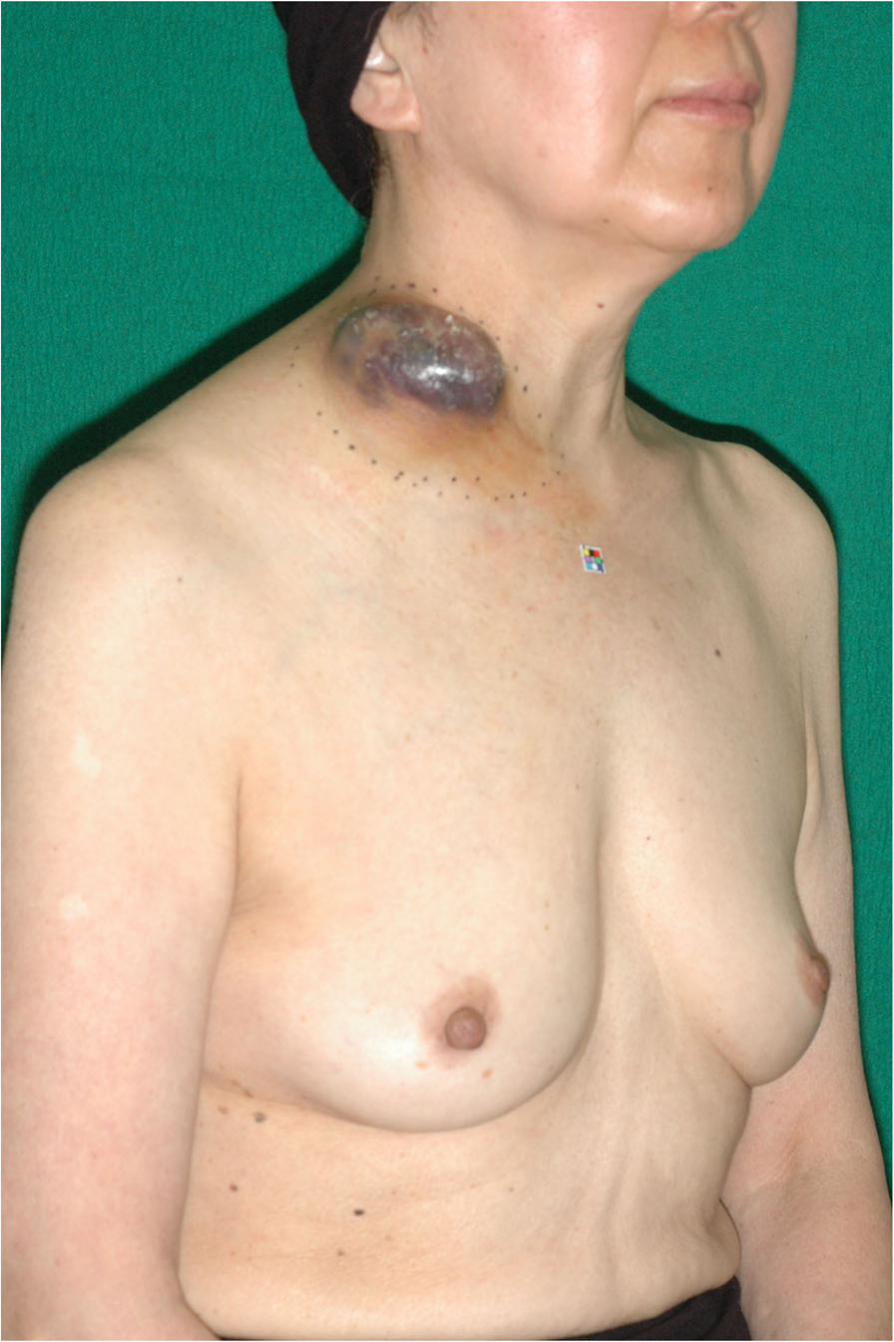
Preoperative status of case 2. A large tumor was observed in the right neck. The size was approximately 10 × 8 cm, based on the marker on the sternal notch

**Fig. 5:**
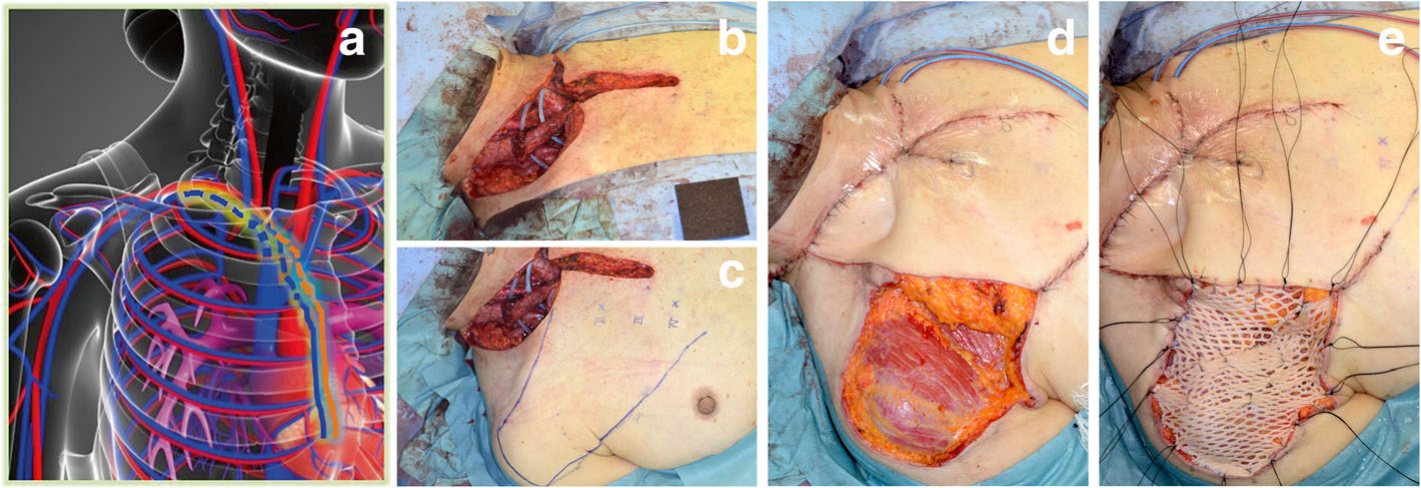
Intraoperative findings in case 2. **a** The schema shows the arteries and veins of the operative site. The interrupted lines show the sacrificed vessels, including the internal thoracic artery and vein, subclavian vein, and brachiocephalic vein. **b** Right anterior oblique view of the operation. The anterior surface of the trachea can be seen, as the thyroid gland had been entirely removed. The right sternocleidomastoid muscle was reconstructed to cover the arteries and veins of the neck while two suction drains were placed under the muscle. **c** The design of the DP flap was drawn in the usual manner. The distal edge was extended to the mid-lateral line of the shoulder. **d** The deltoid muscle and the pectoralis major muscle are observed after DP flap setting. Airtight conditions were ensured to maintain efficacy of the suction drains. **e** Split thickness skin grafting was performed to the donor site of the DP flap. The skin graft had been processed using a mesh dermatome to fit the uneven surface of the muscle and fatty tissue

**Fig. 6:**
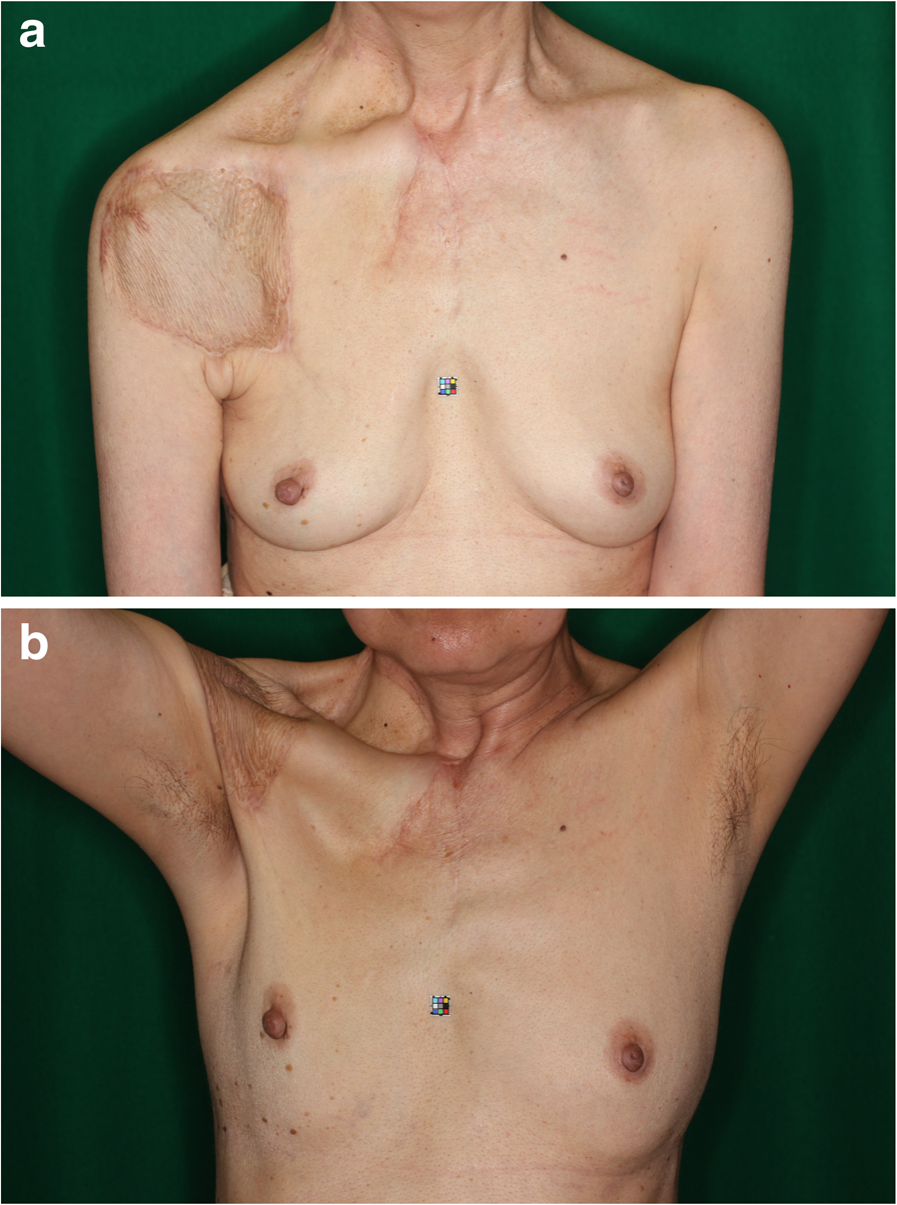
Outcome of case 2 at postoperative 2 years. **a** The flap and the skin graft fit well with the surrounding skin. No obvious scars were observed. **b** The patient can raise her right arm as well as the left arm. There is no derangement of the right shoulder

A 67-year-old woman presented to our hospital with undifferentiated carcinoma of the thyroid gland with local metastasis to neck lymph nodes that was diagnosed based on needle biopsy outside our hospital. The patient presented with no other remarkable symptoms while she had few past medical histories. The tumor size was large enough to raise suspicion of skin involvement during her evaluation at the referring hospital. She was then referred to our hospital due to skin involvement and the need to sacrifice the surrounding large vessels, such as the right common carotid artery and right subclavian artery and vein, during tumor resection and total thyroidectomy (Fig. 4). At first, various flaps such as the DP flap, the pectoralis major myocutaneous flap, the latissimus dorsi flap, and free flaps were considered as candidates for reconstruction of the skin defect. We decided against using a pectoralis major myocutaneous flap because a portion of the right subclavian vein, brachiocephalic vein, and proximal portion of the right internal thoracic artery and vein would have to be sacrificed with dissection of the right neck and paratracheal lymph nodes, while salvaging the right common carotid artery (Fig. 5 a, b). The right DP flap was likely to be excluded as an option before pulsation of the second intercostal perforator artery was confirmed using a Doppler stethoscope. Ultimately, the DP flap was designed and elevated in the usual manner because the 2nd and 3rd intercostal perforator arteries were detected by the Doppler stethoscope. Bleeding from the distal edge of the flap was enough to confirm blood supply of the flap, even after sacrificing the branch of the thoracoacromial artery, and the flap was elevated completely. A portion of the skin on the right side of the neck was undermined and used as a skin flap, followed by setting of the DP flap, which allowed for the airtight closure of the skin defect (Fig. 5 c-e). A portion of the donor site of the DP flap was covered by a meshed skin graft taken from the lower left abdomen. The postoperative clinical course was uneventful to hospital discharge. Because the pathological diagnosis was undifferentiated carcinoma of the thyroid gland, chemoradiotherapy was administered, with no adverse effects. There were no signs of recurrence or metastasis 2 years postoperatively, while the range of motion of the right shoulder improved and no scar contracture developed around the skin graft (Fig. 6, Additional file 2: Figure S2 and Additional file 3: Figure S3).

## Discussion and conclusions

In cases of small sized defects in the neck, direct closure or a small local flap is the first choice for reconstruction, because the skin of the neck is thin and has good extensibility. In patients with thyroid cancer and skin invasion, however, the thyroid gland has to be completely resected with some amount of adjacent skin and paratracheal nodes, because the lesion is classified as stage T4, according to the Unio Internationalis Contra Cancrum. In these cases, additional skin incisions are often made that lead to difficulty in design and elevation of local flaps.

The DP flap was first described by Aymard in 1917 as a method for nasal reconstruction [5]. The history of the DP flap is so old that this flap was regarded a useful candidate for head and neck reconstruction in the 1960s; myocutaneous flaps and vascularized flaps have replaced it in recent years [6]. However, the DP flap is the first choice for closure of neck skin defects because it is thin, pliant, and provides a color and texture match. Moreover, this flap is easy and safe to raise. Its only disadvantage seems to be the need to process the pedicle 2 or 3 weeks after the primary surgery [7, 8]. Skin grafting to the donor site is usually required, but is not always necessary in few cases, as in case 1 reported here.

In cases of neck skin defects caused by radical surgery for the treatment of patients with thyroid cancers, there is little need to perform pedicle treatment as a secondary surgery if DP flaps are applied. Therefore, the DP flap is considered the first choice in most cases of advanced surgery for thyroid cancer, unless the defect is too large [9].

However, blood supply to the flap needs to be taken into consideration. The blood supply of a DP flap is usually provided primarily by the 2nd, 3rd, and 4th intercostal perforating vessels, which arise from the ipsilateral superior costal artery and internal thoracic artery. The internal thoracic artery provides the anterior intercostal arteries in each intercostal space, which connect to the posterior intercostal arteries, the branches of the thoracic aorta, or the supreme intercostal artery. In cases that require sacrificing the proximal ipsilateral internal thoracic artery, arterial blood flow to the DP flap is provided theoretically via bypass of the thoracic aorta. According to some reports, using this flap is contraindicated when the internal thoracic artery has been previously compromised (Fig. 6) [6]. However, if a bypass vessel can supply sufficient blood flow in a short time, the DP flap can be raised safely in the usual manner, namely without the so-called “delay” technique. Based on the clinical course of case 2, our data support this hypothesis, despite the limited number of similar cases in the literature.

In case of radical surgery for late stage thyroid cancer as in case 2, the usual size of the DP flap can be applied to the skin defect even if the ipsilateral internal thoracic artery is sacrificed at its origin. However, it is necessary to confirm survival of the second intercostal perforator artery, which is regarded as the most important nutrient vessel of the DP flap, by Doppler stethoscope. With extreme caution, it is best to confirm bleeding from the distal edge of the flap even after dividing the flap from the branch of the thoracoacromial artery.

The DP flap is still an important reconstructive tool for neck skin defects and has reliable blood supply even after radical surgery for advanced thyroid cancer and other malignant neoplasms of the neck.

## Additional files

Additional file 1: Figure S1. Touch-up surgery for the scar contracture. a: Multiple z-plasty was designed for the scar contracture on the left neck. b: The scar contracture was released after removal of the scar and incisions of the flaps. Each small flap was elevated over the superficial fascia. c: The wound was closed with some trimming of the flap edges. d: Local finding 1 year after multiple z-plasty. The scar contracture was released almost completely although hypertrophic scar was formed again. (ZIP 12226 kb)

Additional file 2: Figure S2. Preoperative reconstructed 3D–CT angiography. a: The left anterior oblique view shows the origin of the left internal thoracic artery (ITA) while the right ITA is observed clearly in the picture. b: In the right anterior oblique view, the arising portion of the right ITA can be seen just behind the yellowish shadow of the tumor in the right neck. The right ITA is clearly seen in this view. (ZIP 1109 kb)

Additional file 3: Figure S3. Postoperative reconstructed 3D–CT angiography. a: The left ITA is located just in front of the aortic arch in this left anterior oblique view. The disconnected portion of the left subclavian artery is due to artifact from the clavicle. The right ITA is quite shallow compared with the left, whereas the arising portion is difficult to visualize. b: In the right anterior oblique view, the right ITA is totally obscured while the left ITA can be seen clearly. (ZIP 512 kb)

## Abbreviations

DP flap: Deltopectoral flap
