# Supplementary figures and images for: Deltopectoral flap revisited for reconstruction surgery in patients with advanced thyroid cancer: a case report
Source: BMC Surg. 2017 Sep 15;17:101. doi: 10.1186/s12893-017-0297-8 (PMC5603086; doi:10.1186/s12893-017-0297-8)

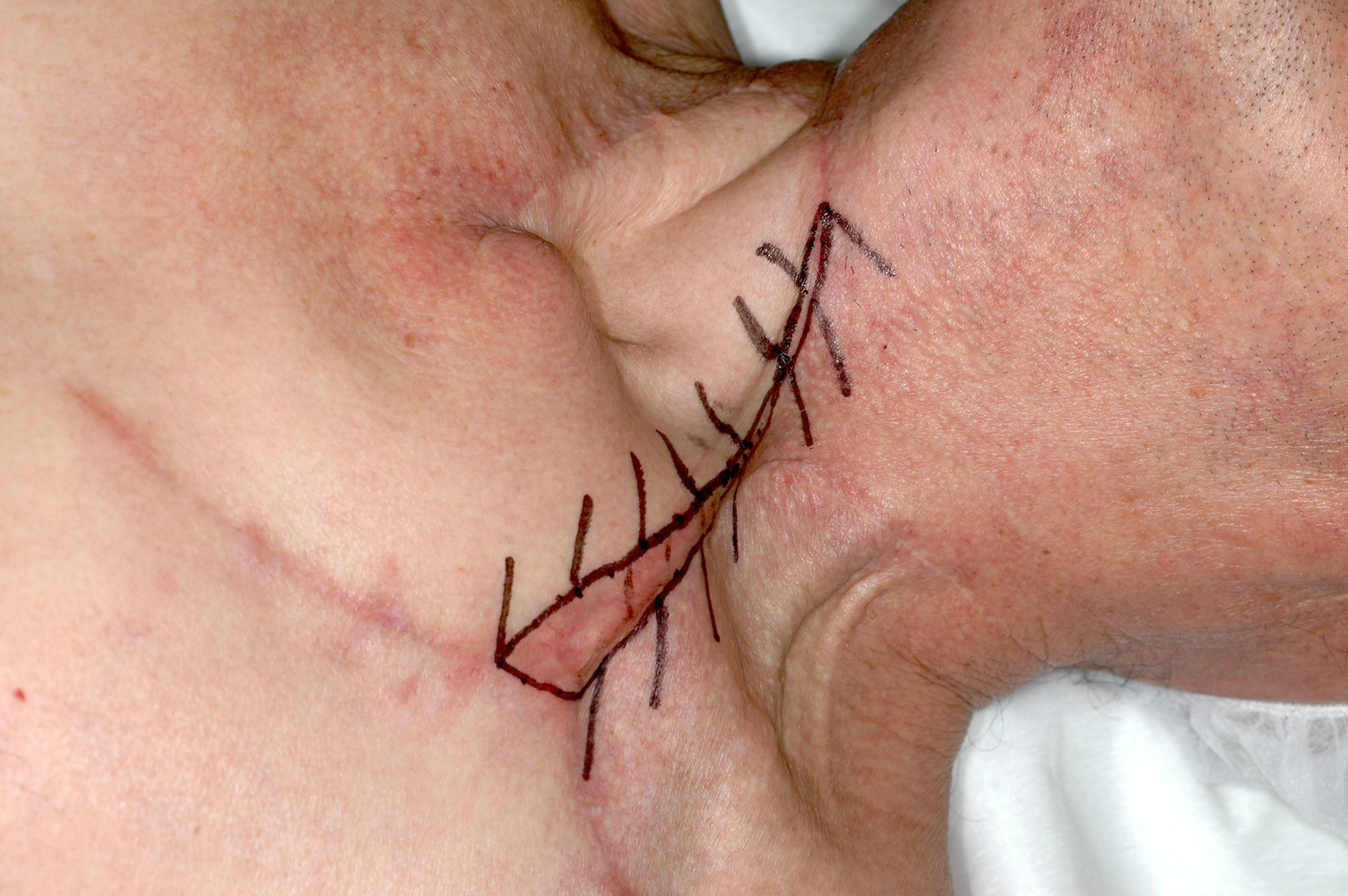

Supplement: Supplementary file 1 — Touch-up surgery for the scar contracture. a: Multiple z-plasty was designed for the scar contracture on the left neck. b: The scar contracture was released after removal of the scar and incisions of the flaps. Each small flap was elevated over the superficial fascia. c: The wound was closed with some trimming of the flap edges. d: Local finding 1 year after multiple z-plasty. The scar contracture was released almost completely although hypertrophic scar was formed again. (ZIP 12226 kb) [file 12893_2017_297_MOESM1_ESM.zip › Mikami. supplemental Fig1aR2.tiff]

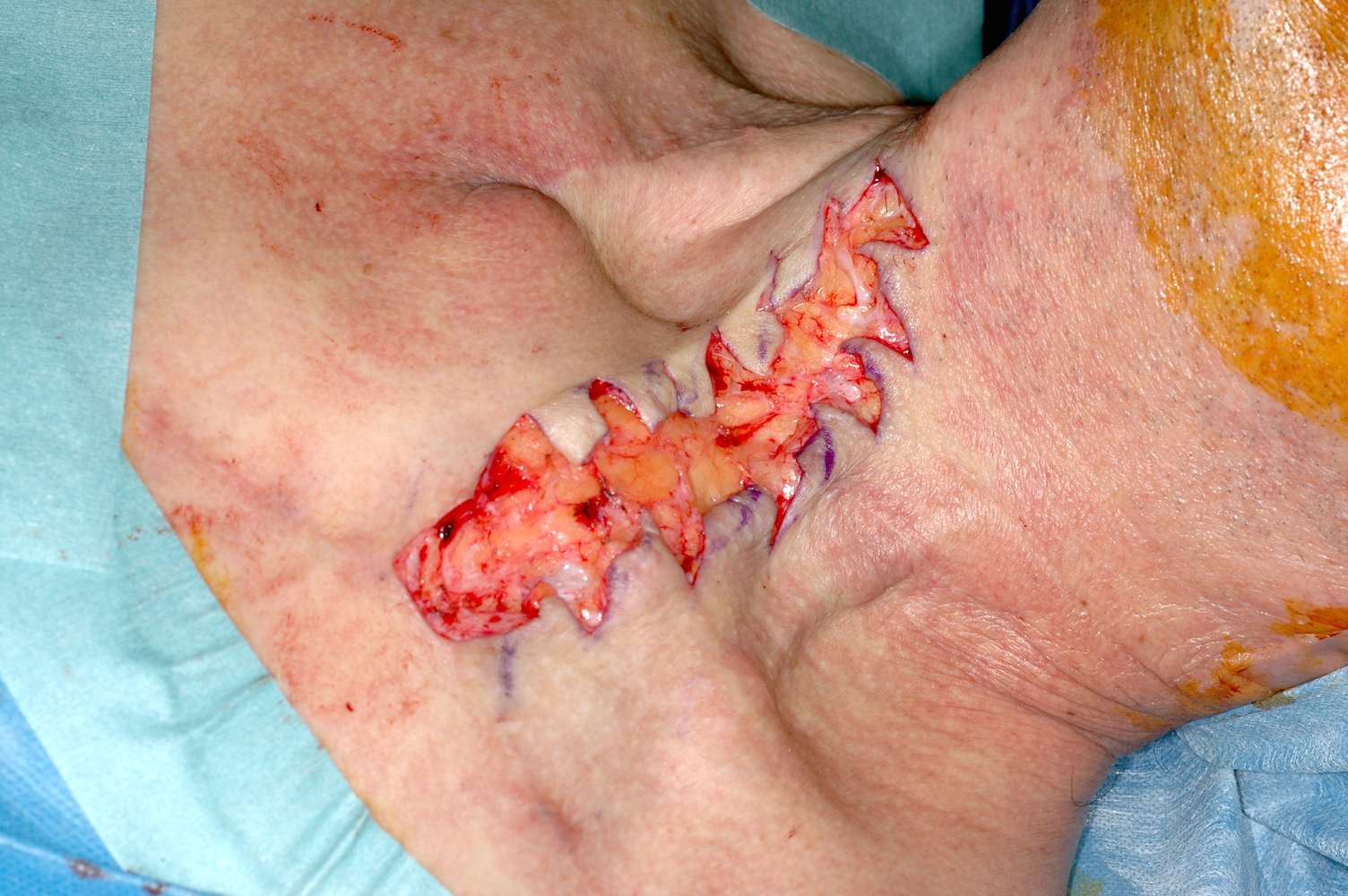

Supplement: Supplementary file 1 — Touch-up surgery for the scar contracture. a: Multiple z-plasty was designed for the scar contracture on the left neck. b: The scar contracture was released after removal of the scar and incisions of the flaps. Each small flap was elevated over the superficial fascia. c: The wound was closed with some trimming of the flap edges. d: Local finding 1 year after multiple z-plasty. The scar contracture was released almost completely although hypertrophic scar was formed again. (ZIP 12226 kb) [file 12893_2017_297_MOESM1_ESM.zip › Mikami. supplemental Fig1bR2.tiff]

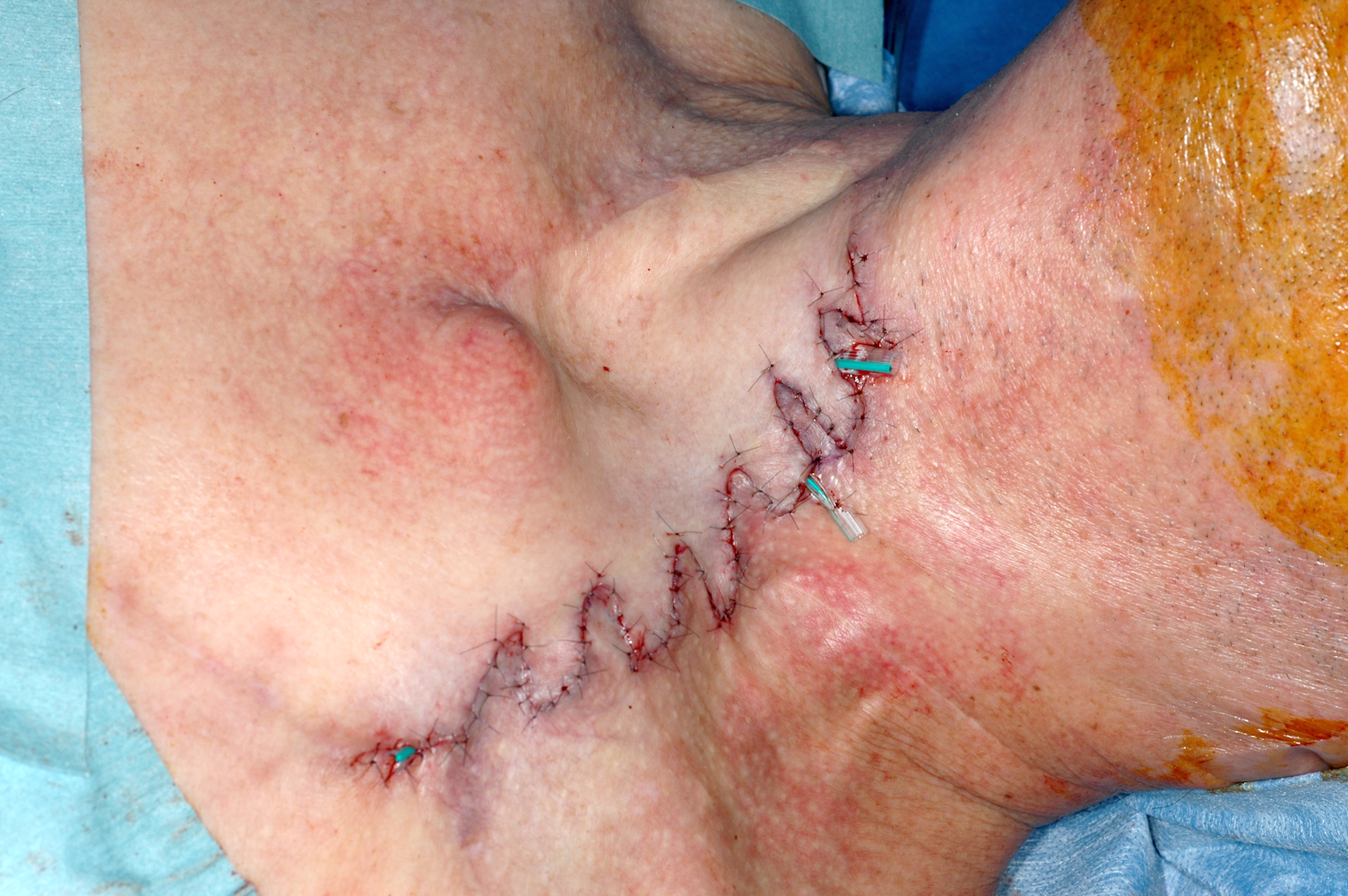

Supplement: Supplementary file 1 — Touch-up surgery for the scar contracture. a: Multiple z-plasty was designed for the scar contracture on the left neck. b: The scar contracture was released after removal of the scar and incisions of the flaps. Each small flap was elevated over the superficial fascia. c: The wound was closed with some trimming of the flap edges. d: Local finding 1 year after multiple z-plasty. The scar contracture was released almost completely although hypertrophic scar was formed again. (ZIP 12226 kb) [file 12893_2017_297_MOESM1_ESM.zip › Mikami. supplemental Fig1cR2.tiff]

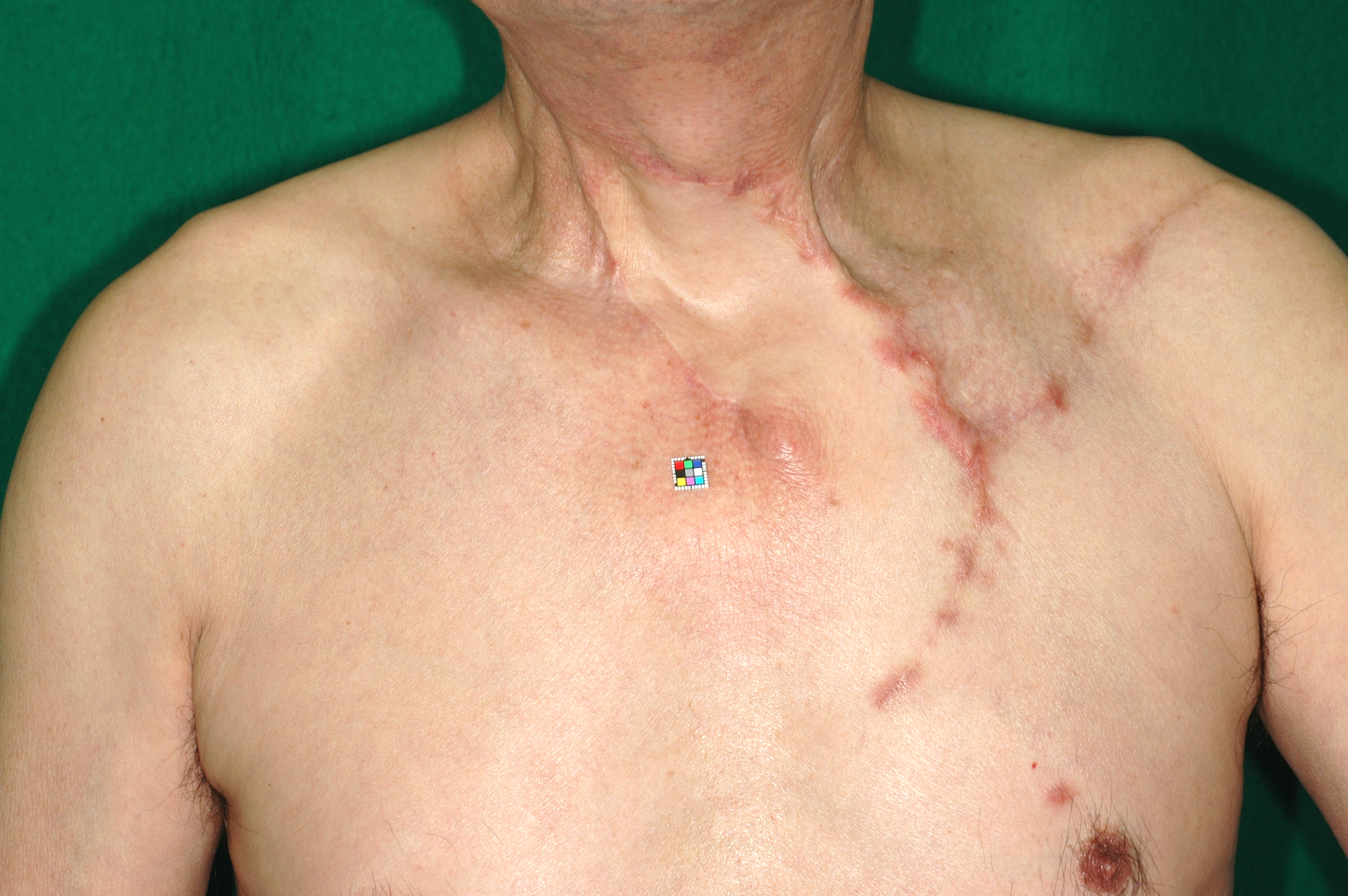

Supplement: Supplementary file 1 — Touch-up surgery for the scar contracture. a: Multiple z-plasty was designed for the scar contracture on the left neck. b: The scar contracture was released after removal of the scar and incisions of the flaps. Each small flap was elevated over the superficial fascia. c: The wound was closed with some trimming of the flap edges. d: Local finding 1 year after multiple z-plasty. The scar contracture was released almost completely although hypertrophic scar was formed again. (ZIP 12226 kb) [file 12893_2017_297_MOESM1_ESM.zip › Mikami. supplemental Fig1dR2.tiff]

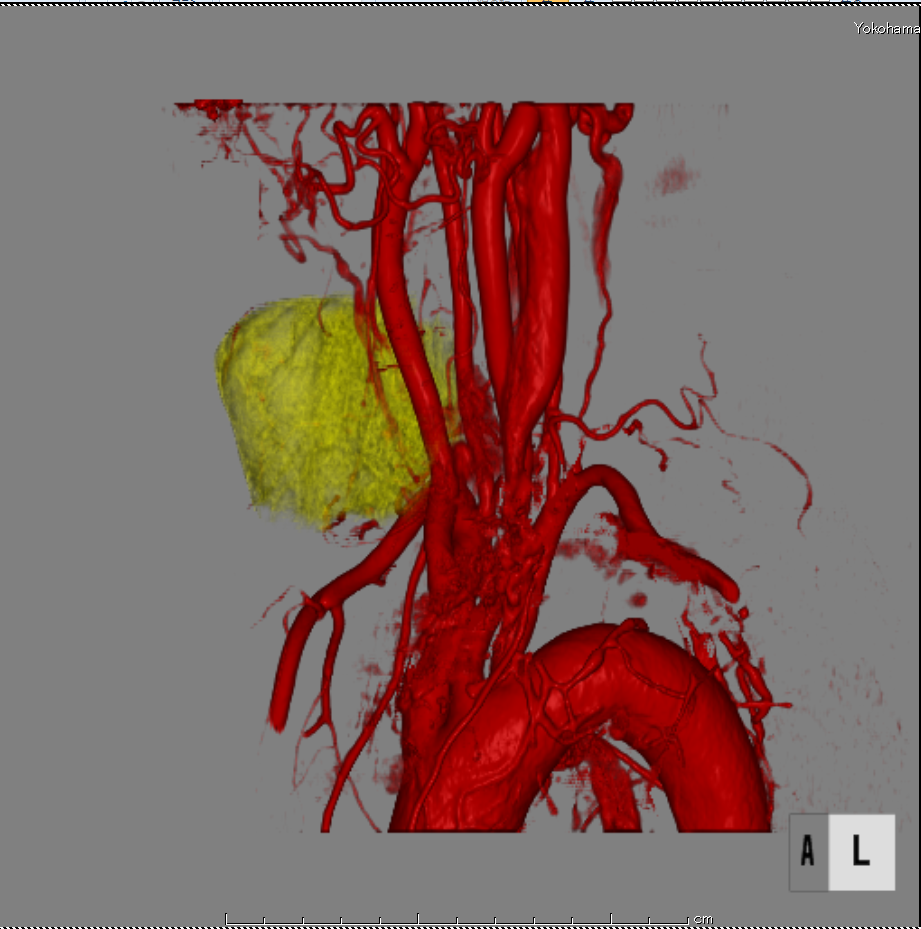

Supplement: Supplementary file 2 — Preoperative reconstructed 3D–CT angiography. a: The left anterior oblique view shows the origin of the left internal thoracic artery (ITA) while the right ITA is observed clearly in the picture. b: In the right anterior oblique view, the arising portion of the right ITA can be seen just behind the yellowish shadow of the tumor in the right neck. The right ITA is clearly seen in this view. (ZIP 1109 kb) [file 12893_2017_297_MOESM2_ESM.zip › Mikami. supplemental Fig2aR2.tif]

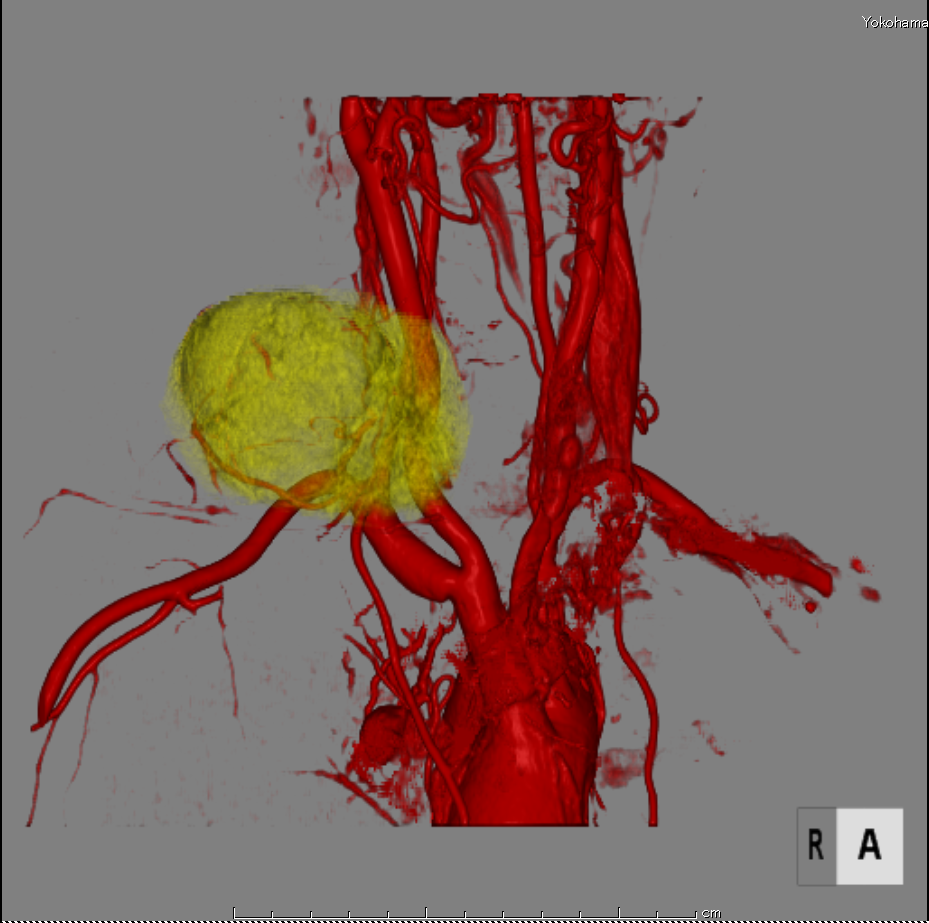

Supplement: Supplementary file 2 — Preoperative reconstructed 3D–CT angiography. a: The left anterior oblique view shows the origin of the left internal thoracic artery (ITA) while the right ITA is observed clearly in the picture. b: In the right anterior oblique view, the arising portion of the right ITA can be seen just behind the yellowish shadow of the tumor in the right neck. The right ITA is clearly seen in this view. (ZIP 1109 kb) [file 12893_2017_297_MOESM2_ESM.zip › Mikami. supplemental Fig2bR2.tif]

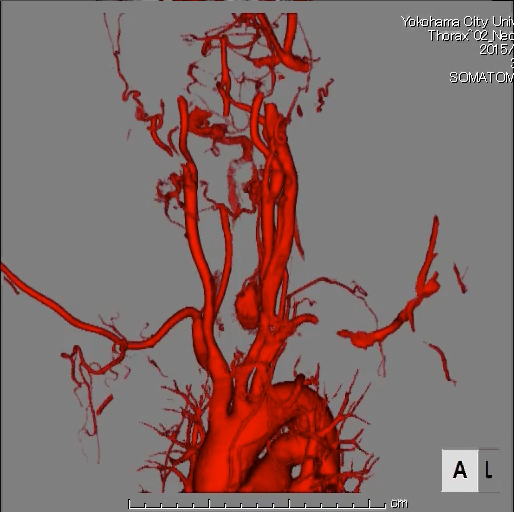

Supplement: Supplementary file 3 — Postoperative reconstructed 3D–CT angiography. a: The left ITA is located just in front of the aortic arch in this left anterior oblique view. The disconnected portion of the left subclavian artery is due to artifact from the clavicle. The right ITA is quite shallow compared with the left, whereas the arising portion is difficult to visualize. b: In the right anterior oblique view, the right ITA is totally obscured while the left ITA can be seen clearly. (ZIP 512 kb) [file 12893_2017_297_MOESM3_ESM.zip › Mikami. supplemental Fig3aR2.tiff]

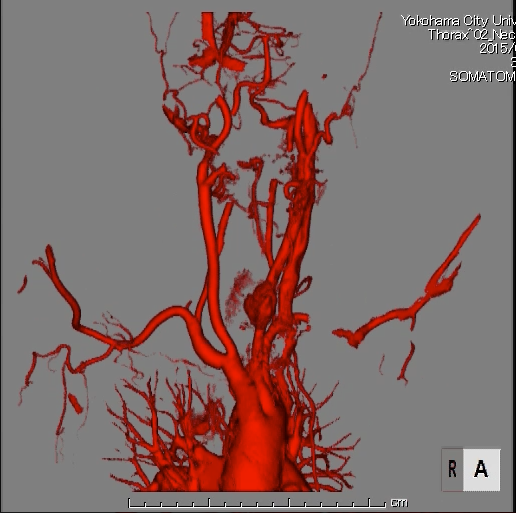

Supplement: Supplementary file 3 — Postoperative reconstructed 3D–CT angiography. a: The left ITA is located just in front of the aortic arch in this left anterior oblique view. The disconnected portion of the left subclavian artery is due to artifact from the clavicle. The right ITA is quite shallow compared with the left, whereas the arising portion is difficult to visualize. b: In the right anterior oblique view, the right ITA is totally obscured while the left ITA can be seen clearly. (ZIP 512 kb) [file 12893_2017_297_MOESM3_ESM.zip › Mikami. supplemental Fig3bR2.tiff]
